# Surface Water Impacted by Rural Activities Induces Genetic Toxicity Related to Recombinagenic Events in Vivo

**DOI:** 10.3390/ijerph13080827

**Published:** 2016-08-16

**Authors:** José Lopes Soares Neto, Raíne Fogliati de Carli, Queila Susana Gambim Kotzal, Francine Bolico Latroni, Mauricio Lehmann, Johnny Ferraz Dias, Cláudia Telles de Souza, Liana Appel Boufleur Niekraszewicz, Fernanda Rabaioli da Silva, Juliana da Silva, Rafael Rodrigues Dihl

**Affiliations:** 1 Laboratory of Genetic Toxicity (TOXIGEN), Post-Graduation Program in Molecular and Cellular Biology Applied to Health, Lutheran University of Brazil (ULBRA), Canoas 92425-900, Brazil; netotocantins@gmail.com (J.L.S.N.); raine_fogliati@hotmail.com (R.F.d.C.); queilak@hotmail.com (Q.S.G.K.); fran_latroni@hotmail.com (F.B.L.); mauriciol@ulbra.br (M.L.); 2 Ion Implantation Laboratory, Institute of Physics, UFRGS, Porto Alegre 91501-970, Brazil; jfdias@if.ufrgs.br (J.F.D.); clau_telles@yahoo.com.br (C.T.d.S.); liana.abn@gmail.com (L.A.B.N.); fer.rabaioli@gmail.com (F.R.d.S.); 3 UniLaSalle, Canoas 92010-000, Brazil; 4 Laboratory of Genetic Toxicology, Post-Graduation Program in Molecular and Cellular Biology Applied to Health, Lutheran University of Brazil (ULBRA), Canoas 92425-900, Brazil; juliana.silva@ulbra.br

**Keywords:** agriculture, genotoxicity, somatic cells, surface water, SMART

## Abstract

This investigation assessed the interaction of surface water samples with DNA to quantitatively and qualitatively characterize their mutagenic and/or recombinagenic activity. Samples were obtained at three different sites along the Tocantins River (Tocantins State, Brazil). The area has withstood the impact mainly of rural activities, which release different chemical compounds in the environment. The *Drosophila melanogaster* Somatic Mutation and Recombination Test (SMART) was performed in standard (ST) and high bioactivation (HB) crosses. SMART is useful for the detection of mutational and recombinational events induced by genotoxins of direct and indirect action. Results demonstrated that samples collected in both seasons were able to induce increments on the mutant spot frequencies in the larvae of the HB cross. Genotoxicity was related to a massive recombinagenic activity. The positive responses ascribed to only the HB cross means that it is linked to pro-genotoxins requiring metabolic activation. The SMART wing test in *Drosophila melanogaster* was shown to be highly sensitive to detect genotoxic agents present in the aquatic environment impacted by agriculture.

## 1. Introduction

Characterized as complex mixtures of various chemical compounds, pesticides are widely used in agriculture to control pests and weeds and for combating plant diseases. Several studies in the specialized literature have discussed the association between exposure to pesticides and increased risk of various cancer types, such as Hodgkin lymphoma [1], leukemia [2], multiplemyeloma [3], in addition to diseases of the immune, nervous, reproductive, and hematological systems [4]. Other dangers of pesticides include the induction of chromosomal mutations [5], sister chromatid exchange [6], and genetic damage [7] in occupationally exposed individuals.

As one of the human activities that systematically release a number of chemical compounds in the environment, the use of agrochemicals in food production may affect not only human health, but also ecosystems, depending on the extent of exposure and contamination [8,9]. Due to the expansion of agriculture in Brazil, the country ranks among the top nations in the use of agrochemicals. For this reason, large amounts of potentially toxic pesticides are discharged on the environment, ultimately reaching watercourses.

Water pollution poses serious environmental hazards, and may become a serious public health problem and a severe issue in aquatic ecosystems [10,11]. Concerning natural water resources, surface water plays an important role in ecosystems, especially due to the propagation of contaminants in the aquatic environment. Therefore, evaluating water quality stands as a source of valuable information about the health status of ecosystems impacted by agriculture.

Lying between the 2nd and the 18° parallels south and meridians 46° and 55° west of Greenwich, the Tocantins River Basin covers a 767,000-km^2^ area exclusive to the Brazilian territory. The river flows south to north 2500 km, crossing the states of Goiás, Tocantins, Maranhão, and Pará, where it drains into the Atlantic Ocean near the Amazon River Estuary [12]. Besides its hydropower potential, the Tocantins River and the power damns built along its bed provide water to soy, maize, and rice plantations in the region, and are used as receptor bodies to agriculture effluent discharges.

In this scenario, the present study evaluated the genotoxicity of surface waters of the Tocantins River impacted by rural activities using the somatic mutation and recombination test in somatic cells of *Drosophila melanogaster* (SMART). The test affords to investigate the loss of heterozygosity (LOH) of marker genes expressed as somatic mutation and recombination after exposure of third instar larvae to complex mixtures [13].

Due to the homology of genetic sequences, *D. melanogaster*, the fruit fly, is the invertebrate model organism that is most closely related with humans [14]. The high sensitivity to toxic substances makes *D. melanogaster* an excellent bioindicator in the detection of environmental pollutants, which is why it is used as a replacement of vertebrate species in toxicity assay [15].

## 2. Materials and Methods

### 2.1. Collection Sites and Physical-Chemical Analysis in Situ

**Figure 1:**
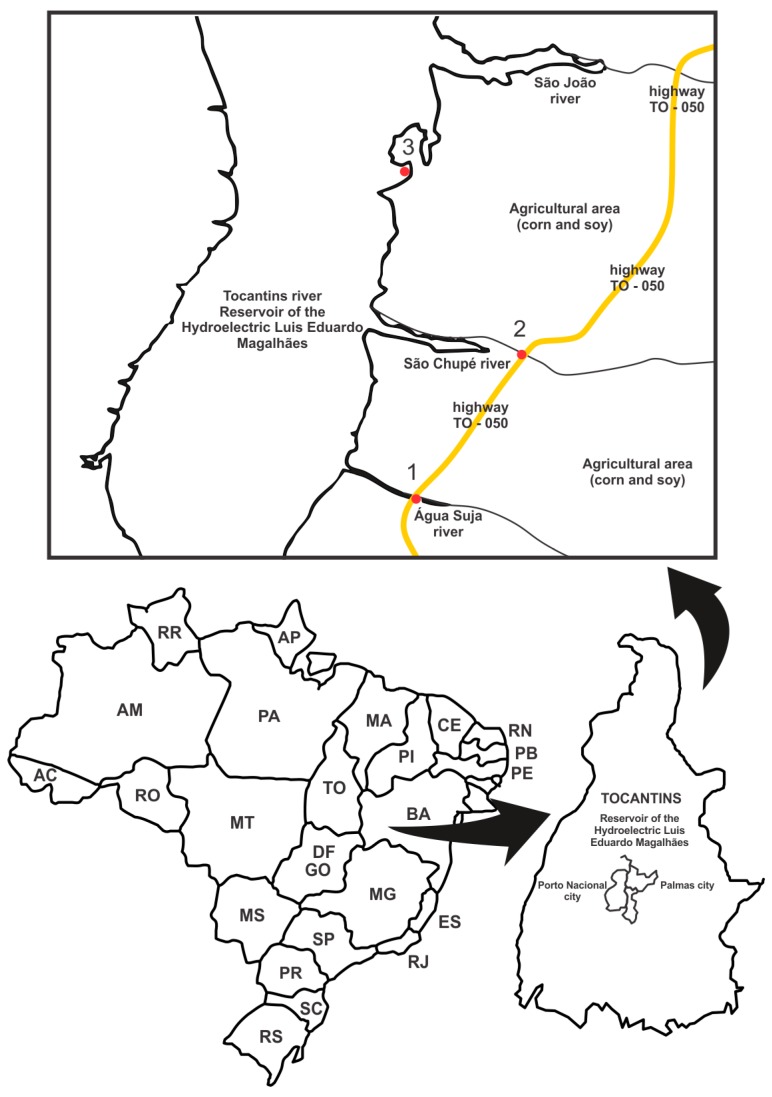
Geographic location of Tocantins River and the map of the collection sites.

Water surface samples were collected at three different sites along the Tocantins River (Figure 1) during two periods, the rainy and dry seasons. Three monthly collections were carried out in the rainy season (November, December 2013 and January 2014) and in the dry season (April, May and June 2013). The data obtained for each site were pooled for season. The water samples were collected and stored following the procedure described by Vargas et al. [16]. The samples were stored for four days at 4 °C, for the quantification of inorganic elements, and then divided into aliquots and kept in a freezer (−20 °C). During the collection, pH, temperature, conductivity, and dissolved oxygen were measured using Mettler Toledo^®^ portable devices. 

### 2.2. Quantification of Inorganic Chemicals

The water samples were analyzed using the Proton Induced X-ray Emission (PIXE) technique. PIXE provides multi-elemental analysis in a straightforward manner by identifying characteristic X-rays emitted from a sample irradiated with a proton beam [17,18]. A 3 MV Tandetron accelerator provided a 2 MeV proton beam with an average current of 3 nA for the irradiation of water samples. In short, the water samples were filtered in membrane filter with 22 μm of thickness. The filters were accommodated in the target holder inside the reaction chamber, which was kept at a pressure of ~10^−6^ mbar. The samples were irradiated for 400 s. The characteristic X-rays induced in the samples were detected using a Si (Li) detector with an energy resolution of approximately 150 eV at 5.9 keV. The PIXE system was calibrated using a range of reference materials. The standardization procedure adopted in this work is described by Johansson et al. [17] and includes all experimental parameters important for the quantitative PIXE analysis. The X-ray spectra were analyzed with the GUPIXWIN software package [19]. The data are expressed as ng/cm^2^.

### 2.3. Somatic Mutation and Recombination Test (SMART)

The *Drosophila* wing SMART provides a rapid means to assess the potential of a genotoxin to induce LOH resulting from gene mutation, somatic recombination, and chromosome breakage or chromosome loss. This bioassay makes use of the wing-cell recessive markers *multiple wing hairs* (*mwh*, 3–0.3) and *flare*^3^ (*flr*^3^, 3–38.8) in trans-heterozygous *mwh +/+ flr*^3^ animals. When a genetic alteration is induced in a mitotically dividing cell of a developing wing disc, it may give rise to a clone(s) of *mwh* and/or *flr*^3^ cells (i.e., a “spot”) visible on the wing surface of the adult fly. The total number of clones induced in a group of chemically treated flies gives quantitative data concerning the whole genotoxic activity of a compound, whereas the types of clone can reveal the mutational mechanisms involved in clone production. Single *flr*^3^ or *mwh* spots (both small and large clones) indicate the occurrence of either a point mutation (in *flr*^+^ or *mwh*^+^), a chromosomal alteration (e.g., a deletion of *flr*^+^ or *mwh*^+^), or somatic recombination. On the other hand, twin spots (i.e., spots of adjacent *flr*^3^ and *mwh* cells) are exclusively derived from somatic recombination. Twin spots therefore give a preliminary indication of the recombinagenic action of a compound. It is also useful to distinguish small single spots (one to two mutant cells) from large single spots (three or more mutant cells); this is because small spots are produced during the last one to two rounds of cell division in the pupa, whereas large spots are produced earlier, during larval feeding. There is also another reason to evaluate small spots separately: genetic deficiencies resulting from chromosomal aberrations most often result in only small clones, regardless of the time of initiation, as the affected cells appear to proliferate slowly if at all [20]. 

For the ST cross, virgin females of the strain *flr*^3^*/In (3LR) TM3*, *Bd^S^* were mated to *mwh* males. For the HB cross, virgin females of the strain *Oregon-R* (*ORR*; *flr*^3^/*In* (*3LR*) *TM3*, *Bd^S^*) were mated to *mwh* males [21,22]. Eggs from both crosses were collected at 25 °C and 60%–80% humidity, in the dark, for 8 h in bottles containing a thick layer of fermenting live baker’s yeast supplemented with sucrose. Three days later, the larvae (72 ± 4 h) were washed out of the bottles with tap water through a meshed stainless steel strainer. Both crosses originated larvae with two different genotypes, namely marker-heterozygous (*mwh +/+ flr*^3^) and balancer-heterozygous (*mwh +/TM3, Bd^s^*). Three days later, the larvae from both the crosses were transferred to vials containing 1.5 g of dry Drosophila Instant Medium (Carolina Biological Supply, Burlington, NC, USA) rehydrated with 5 mL of the test solutions. The larvae were allowed to feed on these media until pupation [20]. 

#### 2.3.1. Wing Spot Analysis

Approximately 10 to 12 days after treatment, the emerging flies were collected from the feeding vials and stored in 70% ethanol. The ST and the HB crosses produce two types of descendants that differ phenotypically, based on the *Bd^S^* marker: (i) wild type wings from marker trans-heterozygous; (ii) heterozygous TM3 wings. Wings of wild type flies of both sexes from the two crosses were mounted on slides using Faure’s solution (30 g gum Arabic, 20 mL glycerol, 50 g chloral hydrate, 50 mL water) and analyzed microscopically [13]. The heterozygous TM3 wings were analyzed when positive statistically responses in the total frequency of spots were obtained in the marker-heterozygous. 

#### 2.3.2. Statistical Evaluation

The frequency of each type of spot (small single, large single or twin) and the total frequency of spots per fly for each water samples were compared pair-wise with the frequency of negative concurrent controls (distilled water) using the Kastenbaum-Bowman test [23] (*p <* 0.05). Because of the weak expression of the *flr*^3^ marker in small clones and its lethality in large clones of mutant cells, only the *mwh* clones (*mwh* single and *mwh* twin spots) were used to calculate clone formation frequencies per 10^5^ cells (n/NC). These values are then employed to estimate the contribution of recombination (R) and mutation (M) to the incidence of total mutant spots per fly in trans-heterozygous genotypes, according to the formulae: R = 1 – {(n/NC in *mwh*/TM3 flies)/(n/NC in *mwh*/*flr*^3^ flies)} × 100; M = 100 − R. The *mwh* clone frequency per fly makes it possible to estimate the induction frequency per cell and per cell division. An appropriate estimation of the induction frequency is obtained if the *mwh* clones per fly frequency is divided by the number of cells (48,800) present in both wings. We use 48,800 (24,400 per wing) instead of 60,000 cells (2 × 30,000 considering both wings), because in screening for wing spots we do not examine all the cells in a wing; there are ~24,400 cells in the wing area we inspect for spots [20].

## 3. Results

Surface water samples from three sites in Tocantins River in the municipalities of Porto Nacional and Palmas, state of Tocantins, Brazil, were evaluated. Table 1 shows the rural activities in each collection site. Soy and maize plantations are the main activity around sites 1 and 2, while site 3 is characterized by fruit orchards. The reason to choose the collection sites used in the present study is linked with the influence of the rural activities in these locations, that is, little urban and industrial influence. Considering that the objective of this study was to characterize the genotoxic profile of water affected by complex mixtures of agrochemicals, the collection sites used presented the appropriate conditions for this purpose. All indicators regarding physicochemical parameters analyzed showed that the water samples analyzed meet the standards defined in specific Brazilian legislation (CONAMA decree 357/2005), in both the dry and rainy seasons (data not shown).

Table 2 shows the results (mean ± standard error) of inorganic compounds levels present in water from the three collection sites analyzed using the PIXE method. Levels of most inorganic elements were comparatively high in the rainy season, when site 1 presented the highest contents of magnesium, aluminum, phosphor, sulfur, potassium, titanium, iron, and zinc. It should be highlighted that elements like silicon and calcium had the highest levels measured in site 3 during the rainy season. Similarly, site 2 had the highest levels of manganese and iron, also in the rainy season.

The results of chronic exposure of ST and HB larvae to water samples from the three sites are shown in Table 3 and Table 4. Frequencies of small single, large, twin, and total spots, which indicate the total genotoxicity of the water collected in the three sites, are given. The frequency of mutant ST and HB clones after exposure to the negative control (distilled water) were similar to the values obtained in previous experiments using the SMART in wings of *D. melanogaster* [24,25]. Two independent experiments were carried out in triplicate, accounting for the analysis of 40 flies for each collection site. Significance of results was based on the frequency of the various spot categories compared with the respective negative control. The positive control (urethane 20 mM) induced significant increases in the frequencies of all spot categories, when values for the HB cross were significantly higher when compared with the values observed for the ST cross. The frequencies of mutant clones from both the ST and HB crosses after exposure to the positive control were similar to previous results [26].

No water sample collected in any of the three sites induced significant increases in the frequency of total spots in ST larvae, compared with the negative control. In fact, the absence of genotoxicity was observed for water samples collected in both the dry and rainy seasons.

On the other hand, in the HB cross, the water samples collected in sites 1 and 2 induced significant increases in the frequencies of total spots in trans-heterozygous flies. Significant differences were observed in both the dry and rainy seasons, compared with the negative control. No significant differences were observed between treatments with water collected in both the dry and rainy seasons and the negative control in the TM3 heterozygous flies. 

The percentages of recombination were approximately 95% and 93% for larvae treated with water from sites 1 and 2, respectively, during the dry season. In addition, percent recombination observed after exposure to water collected during the rainy season in sites 1 and 2 was roughly 89% and 73%.

## 4. Discussion

Quality of water sources is an essential parameter not only in the identification of toxic agents present in aquatic ecosystems, but also in the effort to investigate the cellular and genetic toxicities of environmental samples. The importance of evaluating genotoxicity of surface waters lies in the genetic risk to humans and aquatic systems [29]. In fact, various studies based on different bioassays have shown that river waters in several countries are constantly exposed to contamination by an array of biotoxins present in anthropogenic discharges [11,30,31,32,33].

Aiming to look into the genotoxic condition of surface waters exposed to the influence of agricultural activities, the present study investigated the mutagenic and/or recombinagenic potential of samples collected at three different sites along the Tocantins River between November 2012 and April 2013 (rainy season) and July and September 2013 (dry season). Three collection excursions were carried out to each site, the results thereof being pooled on a season basis.

Exposure of HB larvae to water collected in sites 1 and 2 in both seasons show the genotoxic potential of the samples. Heterozygous TM3 larvae did not indicate genotoxicity of waters from any of the sites. Since the single spots observed in heterozygous TM3 flies reflect predominantly gene and chromosomal mutations, the results obtained show that the samples analyzed induce somatic recombination in proliferative *D. melanogaster* cells. This is due to the fact that the somatic recombination products involving chromosome TM3, with multiple inversions, and its structurally normal homologue are unviable [20]. 

In fact, recombination events were the main mechanism inducing lesions in HB larvae exposed to Tocantins river water in both seasons. Also, these data indicate the existence of genotoxins able to induce lesions associated with recombination between homologous chromosomes, which may produce metabolites that interact with the DNA of somatic cells. The main difference between the ST and HB crosses is the high level of CYP6A2 in the latter. CYP6A2 is similar to the subfamily CYP3A of humans [34]. Studies using the SMART indicated increasing frequencies of homologous recombination in larvae exposed to surface water samples [35].

PIXE results showed that all inorganic elements investigated were detected in the three collection sites in both seasons, except phosphor, chlorine, and manganese. The highest levels of these elements were recorded in the rainy season, possibly due to surface runoffs. In addition to other characteristics, inorganic elements catalyze oxidation reactions, generating reactive oxygen species (ROS) [36]. Genotoxicity and carcinogenicity of several metals have been associated with the induction of oxidative stress. Elements such as aluminum, iron, and magnesium may be genotoxic and cytotoxic, even when present at low concentrations in the environment [37]. Often found in the environment as silicon dioxide (silica) or as silicates, silicon is yet another element with known genotoxicity that has been implicated in the generation of ROS, ultimately causing oxidative damage [38,39]. It should be stressed that oxidation reactions catalyzed by enzymes of the cytochrome P450 group play a significant role in the generation of superoxide anion radicals [37].

Inorganic elements are also present in the formulation of agrochemicals and fertilizers widely used in soy and maize plantations as well as in fruit orchards. Therefore, the levels of genotoxicity observed in the present study may be attributed in part to the presence of agrochemicals in the samples analyzed. Research has shown that the induction of redox signaling may intermediate the toxicologic effects caused by these chemical elements. Exposure to a variety of pesticides induces oxidative stress, which in turn increases levels of ROS, lipid peroxidation, and DNA damage [40]. Importantly, besides enzyme conversion that generates reactive metabolites and/or ROS, pesticides may alter the cell redox status through various other mechanisms, among which the reduction of levels of antioxidants and of the activity of antioxidant enzymes [41].

Significantly high frequencies of spots were observed only for HB larvae, whose metabolic activation is quite high. Research has demonstrated the mutagenic potential of pesticides both in vitro and in vivo [5,42,43]. Recently published findings have demonstrated that pesticides are highly mutagenic mixtures that induce significant increases in micronucleus frequencies in metabolically competent HepG2 cells [44]. Fungicides have been shown to induce genotoxic effects in the isoforms CYP 1A and CYP 2E [45]. The herbicides glyphosate and atrazine have been proved mutagenic to Chinese ovary hamster (CHO) cells, but only when the S9 metabolization fraction was used [46].

Even though pesticides were not quantified in the present study, personal communications with farmers revealed that 17 agrochemicals, including fungicides and insecticides, had been used in the plantations near the collection sites. Some of these agrochemicals were organophosphorus compounds as well as carbamates and pyrethroids. These chemicals are applied as one- or multi-compound formulations during seed preparation stages or sprayed over plantations. One of the most commonly used pesticides is metalaxyl-m, a fungicide whose mutagenic action has been confirmed in human lymphocytes [47]. Propiconazole may induce the expression of different CYP450 isoforms, such as CYP 1A2 in rat liver [48]. In turn, cypermethrin induces the isoform CYP 1A in human hepatocytes [49]. Both compounds have been shown to activate aryl-hydrocarbon receptors in various cell cultures, thereby activating the transcription of different genes of the CYP450 family [50].

The results obtained in the present study show that the surface waters of Tocantins River are genotoxic to somatic cells of *D. melanogaster*. Genotoxicity was observed in both the rainy and dry seasons, only in HB larvae. Water in site 2 collected in both seasons induced the highest frequency of mutant clones. Indeed, site 2 is located downstream site 1, where it receives its own load of agrochemicals in addition to the compounds already identified in site 1. Samples collected in site 3 did not induce genotoxic effects in *D. melanogaster*, possibly because the collection site is located in a reservoir and therefore is exposed to less intense agricultural impact. In addition, soybean is the main produce grown in sites 1 and 2 (Table 1). A previous study used the PIXE technique and showed that higher concentrations of elements like Al, P, and S were detected in cells from soybean farm workers, compared with control individuals [51]. Of the inorganic elements detected in the present study, Al, P, and S were present in samples collected in sites 1 and 2. These elements are part of the formulations of different pesticides and fertilizers, such as carbamates and organophosphates. The same previous investigation also showed that exposure of these workers to complex pesticide mixtures is associated with increased genetic damage [51].

Complex mixtures of inorganic elements, present in pesticides and fertilizers compositions, may have contributed to the high genotoxicity observed in the HB cross. In addition, recombination was the mainly genetic event associated with the formation of mutant clones in flies exposed to sites 1 and 2 in both seasons. The main action mechanism of these compounds is the induction of ROS generation after they have been biotransformed [41]. In this sense, the high levels of P450 enzymes that are characteristic of the HB cross make it more sensitive as an indicator of the lesions caused by the metabolites of these compounds in the SMART. Another relevant aspect is oxidative damage induced by ROS, which significantly induce homologous recombination [52]. The production of ROS is directly correlated with the induction of DNA strand breaks, mainly during metabolization [53]. These lesions are corrected preferentially by somatic recombination. It is known that homologous recombination works as a DNA repair mechanism, which may result in LOH of the genes involved in the regulation of the cell cycle based on the induction of gene conversion, loss of chromosome fragments and translocation, or aberrant genomic rearrangement. It may be said that these events play an essential role in the first stage of carcinogenesis, though they are mostly involved in second and subsequent stages thereof, revealing recessive mutations [52].

## 5. Conclusions

The results obtained in the present study indicate that the surface waters of Tocantins River, Brazil, are contaminated by toxins with indirect action. Considering that the collection sites included in the present study are exposed to the consequences of agricultural activities, pesticides and fertilizers are probably the complex mixture that induced the DNA lesions recorded. The PIXE analysis indicated the presence of inorganic elements, whose levels were higher in the rainy season. Also, all positive samples were shown to induce somatic recombination, expressed mainly as homologous recombination. From this perspective, the SMART is confirmed once again as a sensitive and informative tool to track genotoxicity in surface waters impacted by agricultural activities.

## Figures and Tables

**Table 1:** Contributions of rural activities in each collection site in Tocantins River, Brazil.

Site	Anthropic Influence	Coordinates
1	Soy and maize plantations	10°26′18′′ 48°22′35′′
2	Soy and maize plantations	10°29′45′′ 48°21′05′′
3	Fruit orchards	10°32′02′′ 48°22′37′′

**Table 2:** Inorganic elements detected in the waters collected in sites 1, 2, 3 in Tocantins River, Brazil.

Elements	Rainy Season			Dry Season		
	Collection Sites			Collection Sites		
	Site 1	Site 2	Site 3	Site 1	Site 2	Site 3
Mg	330 ± 25	136± 45	265 ± 28	215 ± 26	104 ± 48	106 ± 46
Al	12,639 ± 4	9511 ± 4	4404 ± 6	8381 ± 3	4385 ± 4	2554 ± 5
Si	135,647 ± 2	91,237 ± 2	137,609 ± 2	41,295 ± 2	34,218 ± 2	40,605 ± 2
P	334 ± 43	98 ± 117	ND	ND	61 ± 133	ND
S	203 ± 24	141 ± 24	19 ± 39	224 ± 31	105 ± 61	98 ± 65
Cl	ND	49 ± 35	135 ± 29	430 ± 17	307 ± 22	315 ± 21
K	560 ± 18	250 ± 28	479 ± 20	389 ± 14	206 ± 21	156 ± 26
Ca	1160 ± 6	1270 ± 6	6286 ± 4	2230 ± 4	2026 ± 4	2090 ± 4
Ti	1784 ± 5	1136 ± 6	393 ± 9	1298 ± 4	1359 ± 4	189 ± 10
Mn	ND	745 ± 4	215 ± 10	ND	88 ± 13	65 ± 15
Fe	26,175 ± 1	34,109 ± 1	8400 ± 1	16,150 ± 1	8952 ± 1	5854 ± 1
Zn	41 ± 33	16 ± 65	16 ± 53	35 ± 25	30 ± 26	14 ± 45

ND = not detected (levels below detection thresholds); Values are presented as means ± SE.

**Table 3:** Fly spot data obtained after exposure of marker heterozygous larvae of *D. melanogaster* to surface water of Tocantins River in ST cross.

Seasons and Genotypes	Sites of Collection and Controls	No. of Flies (N)	Spots per Fly (No. of Spots)/Statistical Diagnosis ^a^				Total Mwh Clones ^c^ (n)	Clone Induction Frequencies (per 10^5^ Cells per Cell Division) ^e^ (n/NC*) ^d,f^
			Small Single Spots ^b^ (1–2 Cells) (m = 2)	Large Single Spots ^b^ (>2 Cells) (m = 5)	Twin Spots (m = 5)	Total Spots (m = 2)		
*Rainy*								
mwh/flr^3^	PC	10	2.80 (28) +	0.80 (08) +	0,00 (00) +	3.60 (36) +	35	7.17 {5.89}
	NC	40	0.60 (24)	0.03 (01)	0.10 (04)	0.73 (29)	25	1.28
	Site 1	40	0.70 (28) −	0.08 (03) i	0.00 (00) −	0.78 (31) −	31	1.59 {0.31}
	Site 2	40	0.75 (30) i	0.13 (05) i	0.03 (01) −	0.90 (36) −	35	1.79 {0.51}
	Site 3	40	0.68 (27) −	0.15 (06) i	0.05 (02) i	0.88 (35) −	33	1.69 {0.41}
*Dry*								
mwh/flr^3^	PC	10	2.80 (28) +	0.80 (08) +	0.00 (00) +	3.60 (36) +	35	7.17 {5.89}
	NC	40	0.60 (24)	0.03 (01)	0.10 (04)	0.73 (29)	25	1.28
	Site 1	40	0.55 (22) −	0.23 (09) +	0.03 (01) −	0.80 (32) −	30	1.54 {0.26}
	Site 2	40	0.63 (25) −	0.20 (08) +	0.10 (04) i	0.93 (37) −	37	1.90 {0.61}
	Site 3	40	0.63 (25) −	0.20 (08) +	0.00 (00) −	0.83 (33) −	32	1.64 {0.36}

^a^ Statistical diagnosis according to Frei and Wurgler [27] +, positive; i, inconclusive; −, negative; m: multiplication factor; significance levels α = β = 0.05; ^b^ including rare flr^3^ single spots; ^c^ considering mwh clones from mwh single and twin spots; ^d^ numbers between keys are induction frequencies corrected for spontaneous incidence estimated from negative controls; ^e^ for calculation see Andrade et al. [20]; ^f^ C = 48.800, i.e., approximate number of cells examined per fly; NC = Negative Control using distilled water. PC = Positive Control using Urethane 20 mM.

**Table 4:** Fly spot data obtained after exposure of marker and balancer heterozygous larvae of *D. melanogaster* to surface water of Tocantins River in HB cross.

Seasons and Genotypes	Sites of Collection and Controls	No. of Flies (N)	Spots per Fly (No. of Spots)/Statistical Diagnosis ^a^				Total Mwh Clones ^c^ (n)	Clone Induction Frequencies (per 10^5^ Cells per Cell Division) ^e^ (n/NC*) ^d,f^	Recombination (%) ^h^	Mutation (%) ^h^
			Small Single Spots ^b^ (1–2 Cells) (m = 2)	Large Single Spots ^b^ (>2 Cells) (m = 5)	Twin Spots (m = 5)	Total Spots (m = 2)				
*Rainy*										
mwh/flr^3^	PC	10	23.10 (231) +	4.10 (41) +	3.00 (30) +	30.20 (302) +	298	61.07 {59.22}		
	NC	40	0.75 (30)	0.15 (06)	0.03 (01)	0.93 (37)	36	1.84		
	Site 1	40	1.08 (43) i	0.20 (08) i	0.08 (03) i	1.35 (54) +	54	2.77 {0.92}	88.89	11.11
	Site 2	40	1.35 (54) +	0.18 (07) i	0.08 (03) i	1.60 (64) +	62	3.18 {1.33}	73.08	26.92
	Site 3	40	0.90 (36) −	0.28 (11) i	0.00 (00) i	1.18 (47) −	47	2.41 {0.56}		
mwh/TM3	PC	10	8.50 (85) +	1.80 (18) +	^g^	10.30 (103) +	103	21.11 {19.83}		
	NC	40	0.63 (25)	0.00 (00)		0.63 (25)	25	1.28		
	Site 1	40	0.63 (25) −	0.05 (02) i		0.68 (27) −	27	1.38 {0.10}		
	Site 2	40	0.75 (30) −	0.05 (02) i		0.80 (32) i	32	1.64 {[0.36}		
*Dry*										
mwh/flr^3^	PC	10	23.10 (231) +	4.10 (41) +	3.00 (30) +	30.20 (302) +	298	61.07 {59.22}		
	NC	40	0.75 (30)	0.15 (06)	0.03 (01)	0.93 (37)	36	1.84		
	Site 1	40	1.18 (47) +	0.18 (07) i	0.03 (01) i	1.38 (55) +	55	2.82 {0.97}	94.74	5.26
	Site 2	40	1.48 (59) +	0.13 (05) i	0.05 (02) i	1.65 (66) +	64	3.28 {1.43}	92.86	7.14
	Site 3	40	1.00 (40) −	0.10 (04) i	0.05 (02) i	1.15 (46) −	46	2.36 {0.51}		
mwh/TM3	PC	10	8.50 (85) +	1.80 (18) +	^g^	10.30 (103) +	103	21.11 {19.83}		
	NC	40	0.63 (25)	0.00 (00)		0.63 (25)	25	1.28		
	Site 1	40	0.90 (36) i	0.00 (00) i		0.90 (36) i	36	1.33 {0.05}		
	Site 2	40	0.68 (27) −	0.00 (00) i		0.68 (27) −	27	1.38 {0.10}		

^a^ Statistical diagnosis according to Frei and Wurgler [27]: +, positive; i, inconclusive; −, negative; m: multiplication factor; significance levels α = β = 0.05; ^b^ including rare flr^3^ single spots; ^c^ considering mwh clones from mwh single and twin spots; ^d^ numbers between keys are induction frequencies corrected for spontaneous incidence estimated from negative controls; ^e^ for calculation see Andrade et al. [20]; ^f^ C = 48.800, i.e., approximate number of cells examined per fly; ^g^ only mwh single spots can be observed in mwh/TM3 heterozygotes as the balancer chromosome TM3 does not carry the flr^3^ mutation; ^h^ Percentage of recombination (R) was calculated according to Frei and Würgler [28]: R = 1 − [(n/NC* in mwh/TM3 flies)/(n/NC* in mwh/flr3 flies)] × 100. Control corrected frequencies were used for these calculations. NC = Negative Control using distilled water. PC = Positive Control using Urethane 20 mM.
